# Maternal vaccination with capsular polysaccharide bioconjugates protects against murine *Klebsiella pneumoniae* neonatal sepsis

**DOI:** 10.1126/sciadv.aeh8222

**Published:** 2026-10-14

**Authors:** Paeton L. Wantuch, Jessica Tung, Lloyd S. Robinson, Isra Darwech, Cory J. Knoot, Christian M. Harding, Drew J. Schwartz, David A. Rosen

**Affiliations:** ^1^ Department of Pediatrics, Division of Infectious Diseases, Washington University School of Medicine, St. Louis, MO 63110, USA.; ^2^ Omniose, St. Louis, MO 63110, USA.; ^3^ Department of Molecular Microbiology, Washington University School of Medicine, St. Louis, MO 63110, USA.; ^4^ Department of Obstetrics and Gynecology, Washington University School of Medicine, St. Louis, MO 63110, USA.; ^5^ Center for Women’s Infectious Disease Research, Washington University School of Medicine, St. Louis, MO 63110, USA.

## Abstract

*Klebsiella pneumoniae* is the leading cause of neonatal sepsis in low- and middle-income countries and the foremost cause of infectious deaths in young children. *K. pneumoniae* isolates are becoming increasingly resistant to antibiotics, creating a great need for additional therapies or preventative strategies. Maternal vaccination serves as an attractive approach to protect from *K. pneumoniae* neonatal sepsis. Herein, we report a murine model of maternal immunization followed by pup challenge to evaluate putative *K. pneumoniae* vaccines. Dams were immunized with capsular polysaccharide bioconjugate vaccines at mating and boosted with two different immunization schedules throughout the gestational period. After parturition, offspring were tested for antigen-specific antibody levels and later challenged with a lethal dose of *K. pneumoniae.* Pups born to dams immunized with K1- and K2-conjugate vaccines demonstrated significant protection from sepsis relative to control pups. Antibodies transferred from vaccinated dams persisted and remained protective in pups for at least six weeks after birth. Collectively these results demonstrate a feasible and reproducible murine model of maternal immunization verifying the efficacy of bioconjugate vaccines in protecting against lethal neonatal *K. pneumoniae* infection.

## INTRODUCTION

*Klebsiella pneumoniae* is a Gram-negative opportunistic pathogen capable of causing infection in a variety of host niches (*1*). Perhaps the most concerning disease caused by *K. pneumoniae* is neonatal sepsis. The Burden of Antibiotic Resistance in Neonates from Developing Societies (BARNARDS) study found *K. pneumoniae* to be the leading cause of neonatal sepsis in low- and middle-income countries (*2*). Another such study, the Child Health and Mortality Prevalence Surveillance (CHAMPS), found that *K. pneumoniae* neonatal sepsis was the leading cause of death in children under the age of five in low-and middle-income countries (*3*). Alarmingly, a large number of *K. pneumoniae* isolates encode a variety of antibiotic resistance determinants. In the aforementioned study, 85% of all isolates were found to harbor extended-spectrum beta-lactamase genes (*2*). Further, carbapenem-resistant *K. pneumoniae* affects 0.3% of infants in neonatal intensive care units and can carry up to 25% mortality (*4*). As antibiotic resistance accelerates and current antibiotics fail to protect vulnerable neonates, it is evident that new therapies or preventative strategies targeting *K. pneumoniae* are desperately needed.

Vaccination has long been an exemplary solution to targeting bacterial pathogens without the need for antibiotics. A recent Bayesian model suggested that a maternal vaccine against *K. pneumoniae* could avert 80,000 to 400,000 neonatal deaths annually (*5*). Additionally, the World Health Organization released a vaccine value profile outlining the dire need and ideal characteristics of a *K. pneumoniae* maternal vaccine (*6*). As with other bacterial pathogens, targeting a surface polysaccharide, such as capsule, in conjugation with a carrier protein, is a leading strategy for vaccine design. Recent work has demonstrated that vaccines targeting the capsule of *K. pneumoniae* can prevent infection with homologous serotypes in non-human primate and murine models (*7*–*11*).

Modeling a disease state in preclinical animal models is an important step in evaluating vaccine efficacy. Indeed, murine models have been used to study neonatal sepsis and maternal immunization against group B *Streptococcus* and *Escherichia coli* (*12*–*15*). Specifically for *K. pneumoniae*, two recent studies modeled neonatal sepsis using distinct methods and strains of this pathogen (*16*, *17*). One of these studies leveraged oral administration of a hypervirulent *K. pneumoniae* to neonatal mice (*16*), while another infected female mice with *K. pneumoniae* and then ultimately assessed passage of antibodies to subsequent pups (*17*). Together, these studies demonstrate the promise of a maternal vaccine to target *K. pneumoniae* and the feasibility of using a murine model to demonstrate vaccine efficacy. Virtually all neonatal sepsis infections are thought to be caused by classical (as opposed to hypervirulent) strains of *K. pneumoniae* (*2*, *18*). Therefore, a tremendous need still exists for a novel murine model to evaluate maternal vaccine efficacy using active maternal immunization and clinically relevant classical isolates of *K. pneumoniae*.

Herein, we describe the first neonatal sepsis model to evaluate active maternal vaccination with two capsular polysaccharide bioconjugate vaccines against *K. pneumoniae*. Two classical isolates of *K. pneumoniae* were utilized: one producing K1 capsule and the other producing K2 capsule. We evaluated two distinct bioconjugate vaccines each with two different immunization schedules. We assessed the antibody production in pregnant dams, placental transfer to pups, and functional longevity of these antibodies in the offspring. Finally, we measured the ability of the maternal vaccination to protect offspring from lethal *K. pneumoniae* sepsis challenge. This novel murine model of maternal immunization followed by pup challenge will inform vaccine design and serve to ascertain the efficacy of current and future vaccine candidates in protecting against *K. pneumoniae* neonatal sepsis.

## RESULTS

### Maternal immunization yields robust IgG titers in dams

Bioconjugate vaccines targeting the capsular polysaccharide of *K. pneumoniae* were tested for their ability to generate a robust immune response in pregnant mice. Groups of CD-1 outbred female mice were immunized with either K1-EPA, K2-EPA, or EPA alone as a carrier protein control. These specific vaccines were chosen as they were previously produced, characterized, and available for murine infections (*10*). All vaccines were formulated with 2% aluminum hydroxide gel as adjuvant and contained 1 μg polysaccharide per dose. Prior to immunizations, murine stool was collected and shotgun metagenomically sequenced to confirm that mice were not gastrointestinally colonized with *K. pneumoniae* (fig. S1). All dams were immunized on day 0 at time of mating with males and continued with immunization following a 2-dose (days 0 and 14) or 3-dose (days 0, 7, and 14) schedule (Fig. 1A). Dams were bled over the course of the immunization to obtain serum samples. Sera were analyzed on ELISA plates coated with glycoengineered *E. coli* expressing either the K1 or K2 capsule of *K. pneumoniae* on its surface. There were notable capsule-specific antibodies produced from both K1-EPA and K2-EPA immunization that increased over the course of the experiment (Fig. 1, B to E). Both the 2-dose and 3-dose vaccine regimens generated detectable IgGs in all mice tested by day 21.

**Fig. 1.**
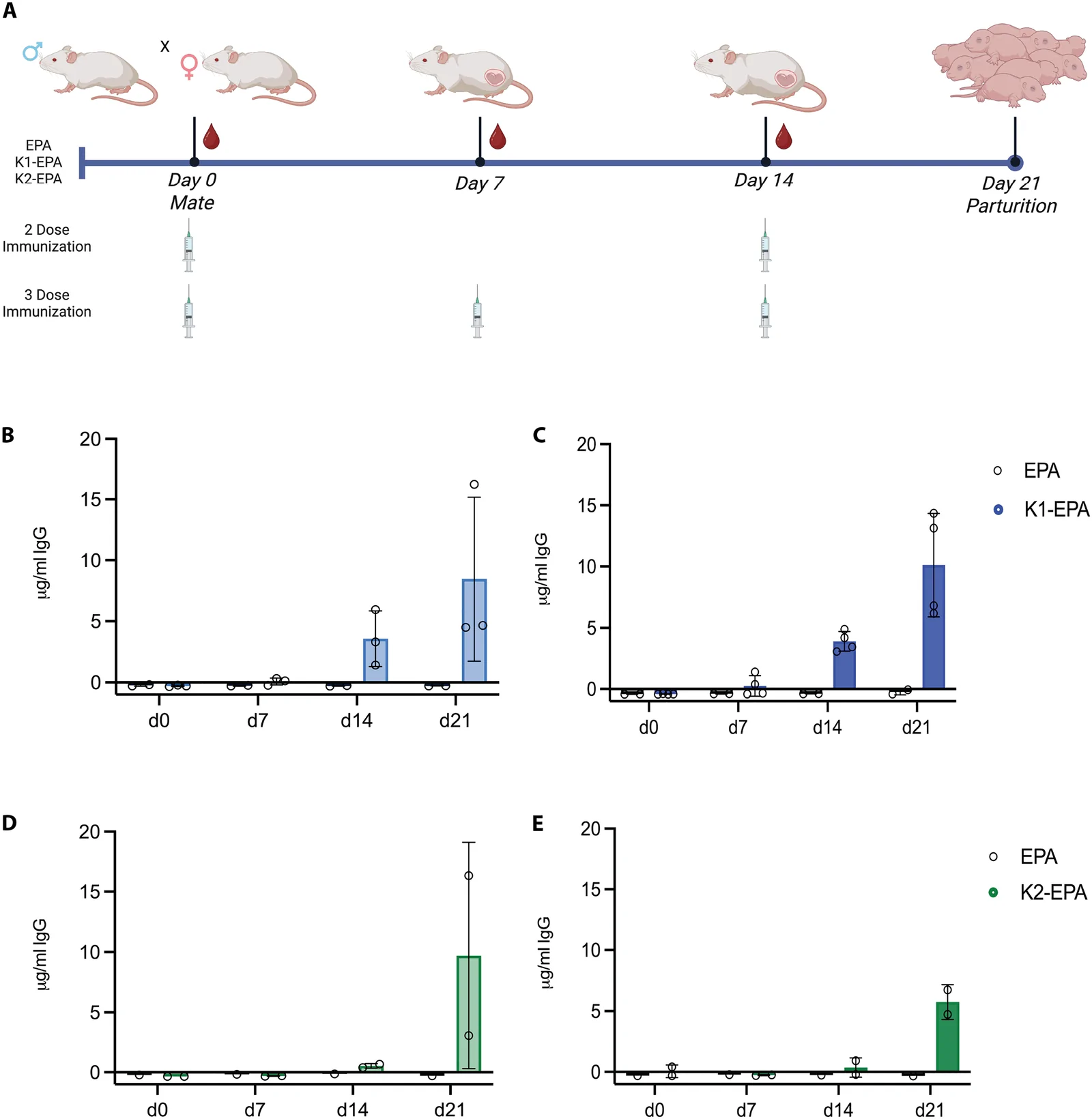
Maternal immunization using K1 or K2 capsule conjugate vaccines. (**A**) Schematic of CD-1 mouse breeding and immunization schedule. Sera was collected from dams throughout the course of immunization to assess specific IgG concentrations as measured by ELISA against glycoengineered *E. coli* expressing (**B** and **C**) K1 polysaccharide or (**D** and **E**) K2 polysaccharide. Dams were immunized with either 2 doses [(B) and (D)] or 3 doses [(C) and (E)] of K1-EPA or K2-EPA conjugate vaccines. Graphs display immunoglobulin concentrations as calculated from a standard curve after background subtraction. Error bars represent standard deviation. Dam numbers are *n* = 1–4 per group. Blue and green legends refer to (B) and (C) and (D) and (E), respectively. (A) was created in created in BioRender. P. Wantuch (2026) https://BioRender.com/v45us2s.

### Maternal immunization results in significant antibody transfer to offspring

Parturition occurred around day 21 in all groups and pup serum was obtained on days indicated up to six weeks of life (Fig. 2). Pup sera were analyzed on ELISA plates coated with K1- or K2-glycoengineered *E. coli* to measure polysaccharide-specific IgG maternally transferred to the pups. Pups born to immunized dams demonstrated significant levels of polysaccharide-specific IgG through 6 weeks of age. Pups born to dams immunized with the 2-dose K1-EPA schedule (Fig. 2A) exhibited approximately 14 μg/ml for the two weeks of life, compared to 20 μg/ml for the 3-dose schedule, followed by subsequent gradual decreases in polysaccharide-specific IgG. Similarly for the K2-EPA immunizations (Fig. 2B), the 3-dose schedule resulted in initially higher polysaccharide-specific IgG concentrations of 10 μg/ml compared to 5 μg/ml for the 2-dose regimen. Further, persistently increased levels of IgG were observed for a longer duration in pups born to dams that received a 3-dose regimen compared to pups born from the 2-dose regimen, although levels remain detectable even in pups born from the 2-dose regimen. Taken together, these data demonstrate that polysaccharide-specific IgG antibodies are detected in offspring of dams immunized with 2 or 3 doses of conjugate vaccine and that transferred antibodies may persist longer in pups born to dams vaccinated with the 3-dose regimen.

**Fig. 2.**
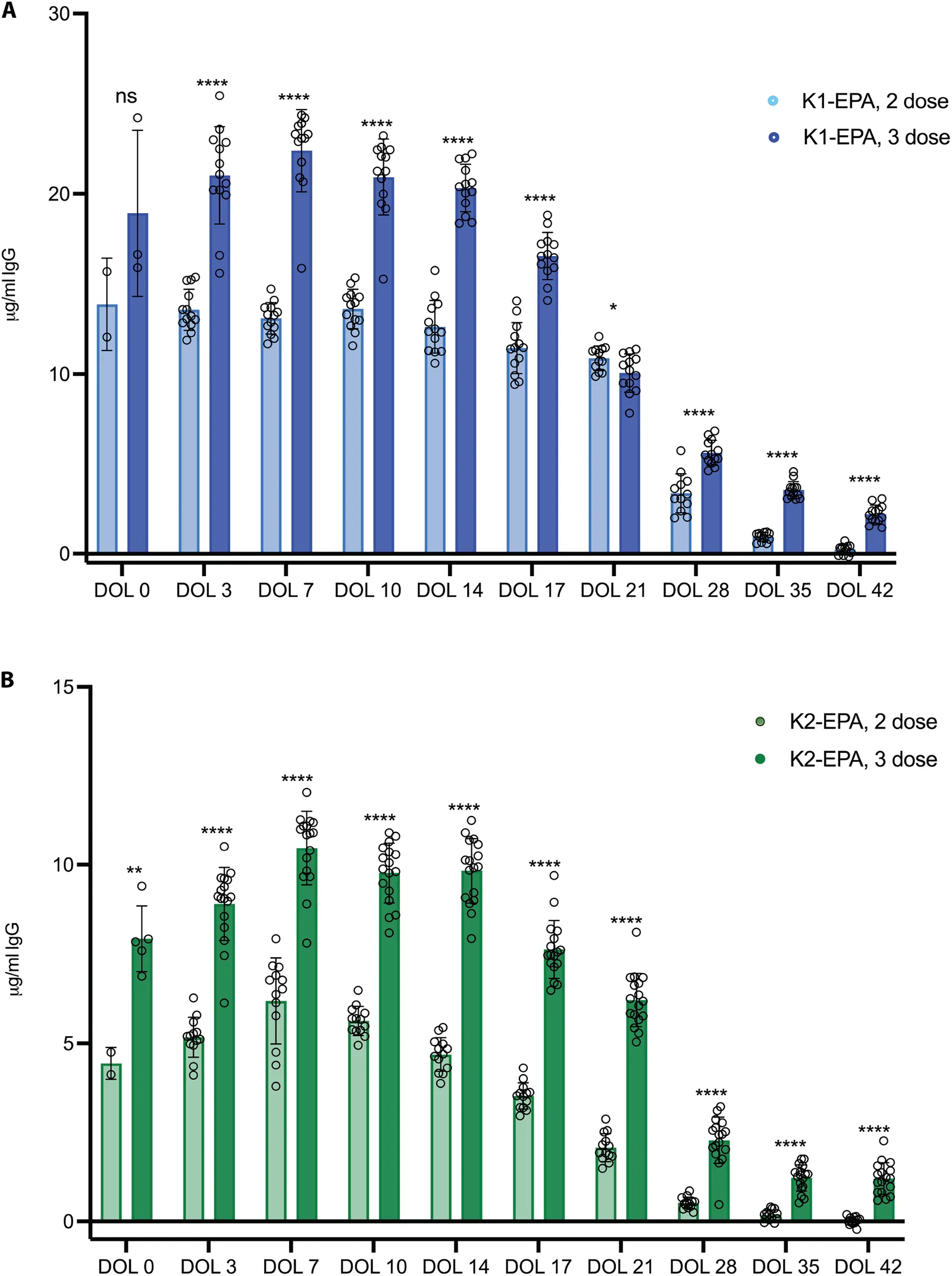
Offspring antibody levels over time. Pups were born to dams immunized with either K1-EPA or K2-EPA conjugate vaccines. Pups were bled on days indicated to measure polysaccharide-specific IgG concentrations as assessed via ELISA using plates coated with glycoengineered *E. coli* expressing (**A**) K1 polysaccharide or (**B**) K2 polysaccharide. Graphs display immunoglobulin concentrations as calculated from a standard curve after background subtraction. Error bars represent standard deviation. Statistics were performed using Welch’s *t* test comparing 2-dose regimen to 3-dose regimen. Pup numbers are *n* = 12–17 per group. *P < 0.05, **P < 0.01, ****P < 0.0001; ns, not significant.

### Maternal immunization generates capsule-specific antibodies in breastmilk

Antibodies can be transferred to offspring through vertical transmission prenatally via the placenta or postnatally through breastmilk. To this end, dam breastmilk was collected around day of life (DOL) 8 of the pups. Breastmilk was tested for both polysaccharide-specific IgA and IgG antibody levels. No detectable polysaccharide-specific IgA was detected in the dam breastmilk (fig. S2). However, significant levels of capsule-specific IgGs were observed in breastmilk of dams vaccinated with bioconjugate vaccines (Fig. 3).

**Fig. 3.**
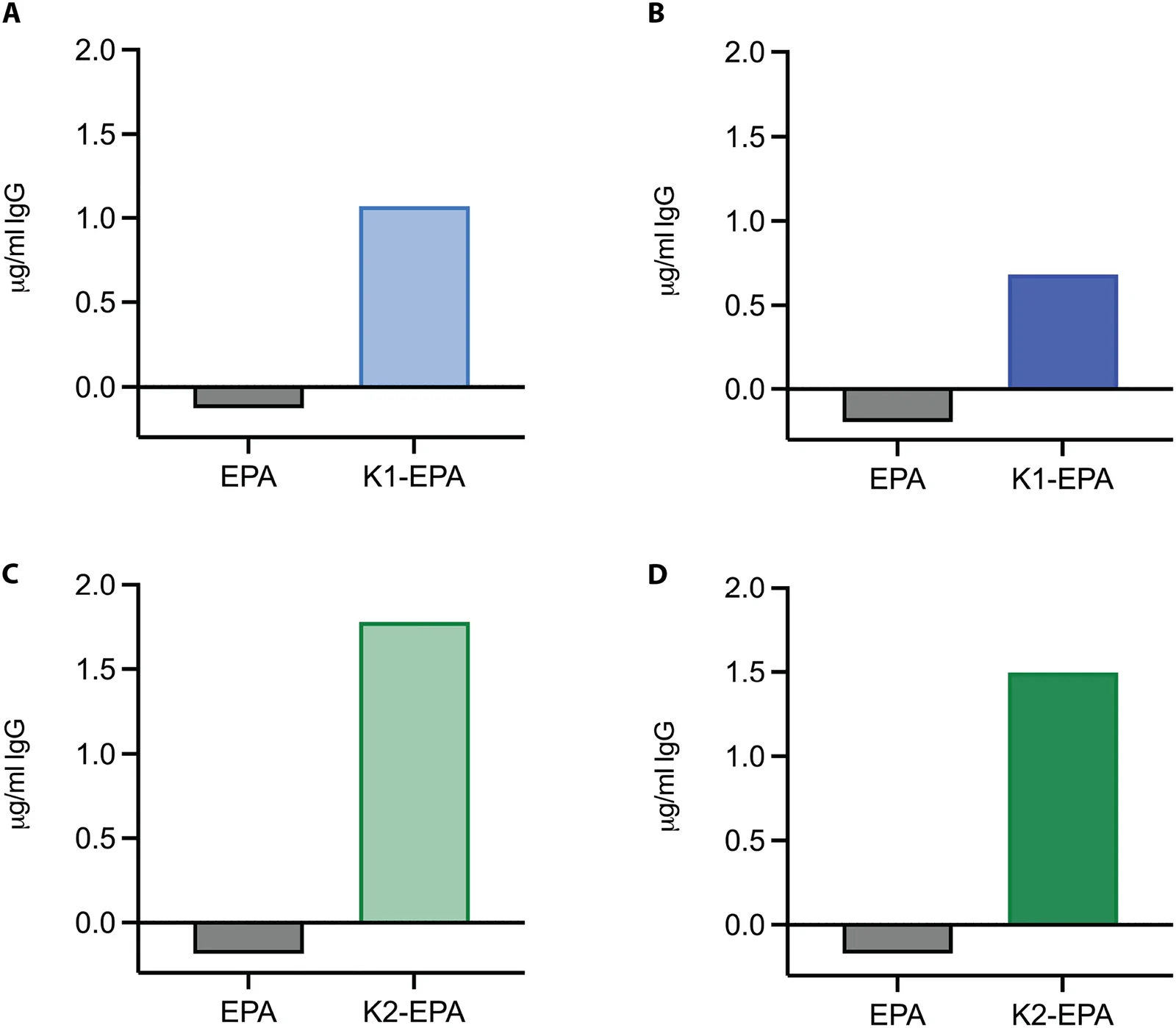
Polysaccharide-specific antibodies in the breastmilk. Breastmilk was collected from lactating dams after immunization with either K1-EPA (blue) or K2-EPA (green) conjugates. Polysaccharide-specific IgG concentrations were detected as measured by ELISA on plates coated with glycoengineered *E. coli* expressing (**A** and **B**) K1 polysaccharide or (**C** and **D**) K2 polysaccharide. Dams were immunized with either 2 doses [(A) and (C), light blue or green] or 3 doses [(B) and (D), dark blue or green] of K1-EPA, K2-EPA conjugate vaccines or EPA (black) as control. Graphs display immunoglobulin concentrations as calculated from a standard curve after background subtraction. Dam numbers are *n* = 1–2 per group.

### Transferred antibodies protect pups from *K. pneumoniae* neonatal sepsis

After establishing that polysaccharide-specific IgG antibodies were transferred to the offspring after maternal immunization, we wanted to test whether these antibodies would be functional and protect pups from lethal bacteremia challenge. First, we established that intraperitoneal injection of either a K1- (cKp120) or K2- (KR174) expressing strain of *K. pneumoniae* led to robust bacteremia in naïve DOL14 pups (fig. S3). We then challenged pups born to immunized dams and found that pups born to K1-EPA-immunized dams were significantly protected from lethal challenge compared to pups born to control-immunized dams regardless of if the dam was immunized 2 or 3 times (Fig. 4, A and B). Infections with the K1 isolate resulted in universal lethality in pups born to dams vaccinated with control vaccine compared to >80% survival in all pups born to K1-EPA-vaccinated dams. Similarly, pups born to dams immunized with K2-EPA exhibited >80% protection from infection with the K2 strain compared to <35% protection observed in pups born to control immunized dams regardless of immunization schedule (Fig. 4, C and D).

**Fig. 4.**
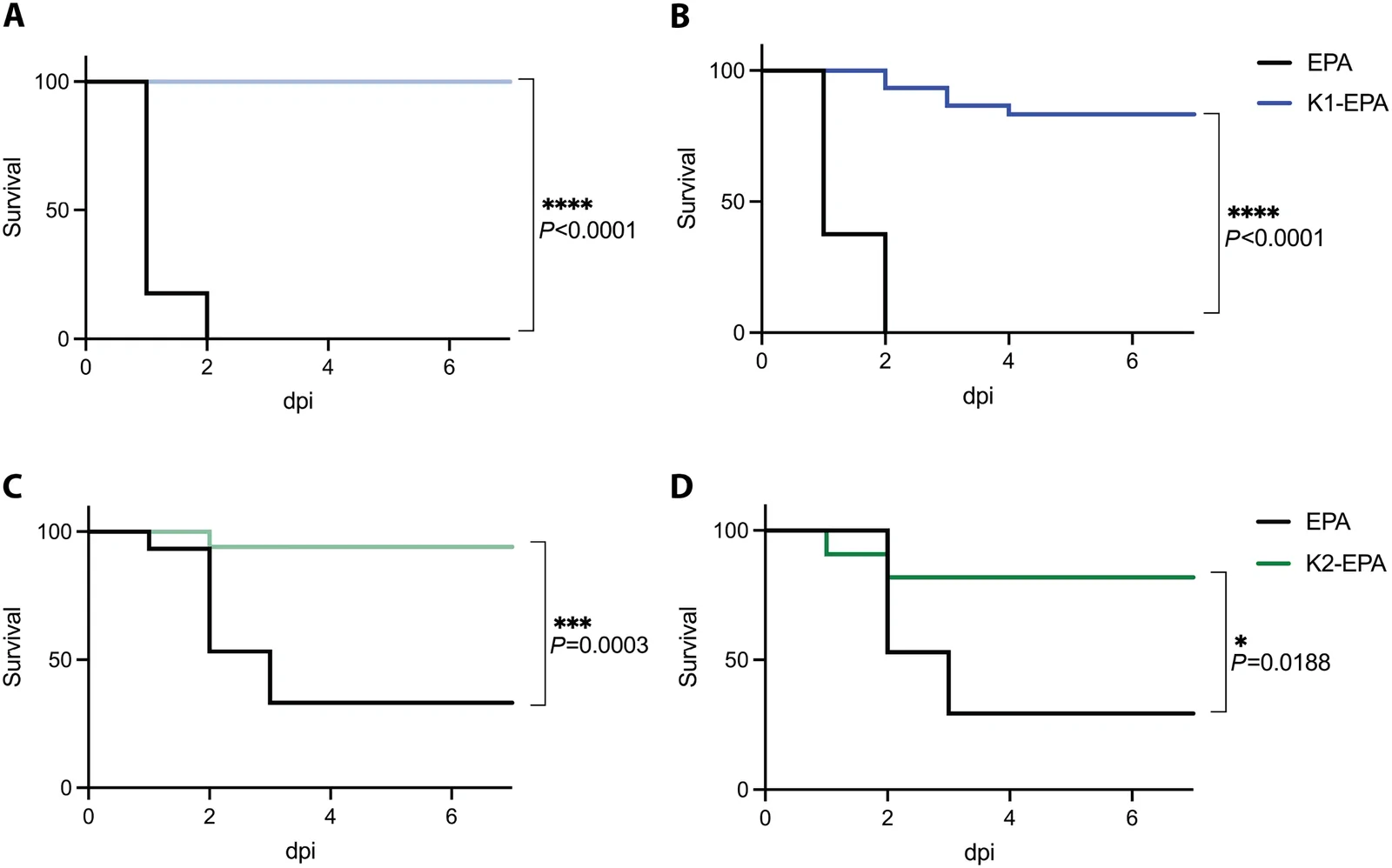
Neonatal offspring are protected from lethal challenge. After parturition, pups were challenged with lethal doses of *Klebsiella pneumoniae*. (**A** and **B**) Pups born to dams immunized with K1-EPA were challenged with cKp120. (A) Pups born to dams immunized on the 2-dose regimen. (B) Pups born to dams immunized on the 3-dose regimen. (**C** and **D**) Pups born to dams immunized with K2-EPA were challenged with KR174. (C) Pups born to dams immunized on the 2-dose regimen. (D) Pups born to dams immunized on the 3-dose regimen. All pups were challenged at day-of-life (DOL) 14. Statistical analyses were performed via log-rank (Mantel-Cox) test comparing pups born to dams immunized with EPA control compared to pups born to dams immunized with K1-EPA or K2-EPA. dpi, days post infection. Pup numbers for K1 studies are *n* = 16–30 per group and pup number for K2 studies are *n* = 11–17 per group. *P < 0.05, ***P < 0.001,****P < 0.0001. Legend beside (B) applies to (A) and (B), and legend beside (D) applies to (C) and (D).

As the ELISA data suggested that maternal vaccine-specific antibodies remained in pup circulation for several weeks after birth (Fig. 2), we tested whether this same cohort of offspring born to immunized dams were still protected from *K. pneumoniae* challenge at 6 weeks post-birth with either cKp120 or KR174. Naïve CD-1 outbred female mice were used as controls in this challenge. Six-week-old mice born to K1-EPA immunized dams on the 2 and 3 dose regimens were 100% protected from lethal bacteremia challenge compared to 40% and 60% naïve CD-1 mice, respectively (Fig. 5, A and B). Pups born to dams immunized 3 times with the K2-EPA vaccine were 100% protected from lethal bacteremia challenge with KR174 at 6 weeks of life compared to 50% of naïve CD-1 mice (Fig. 5D). However, offspring from dams immunized twice with K2-EPA exhibited ∼70% protection from infection compared to 30% of naïve mice; while not significant, these data trend toward protection (*P* = 0.0597, Fig. 5C). Taken together, these data suggest that offspring are protected from *K. pneumoniae* lethal challenge for at least 6 weeks following maternal immunization with a K1 or K2 capsular polysaccharide conjugate vaccine.

**Fig. 5.**
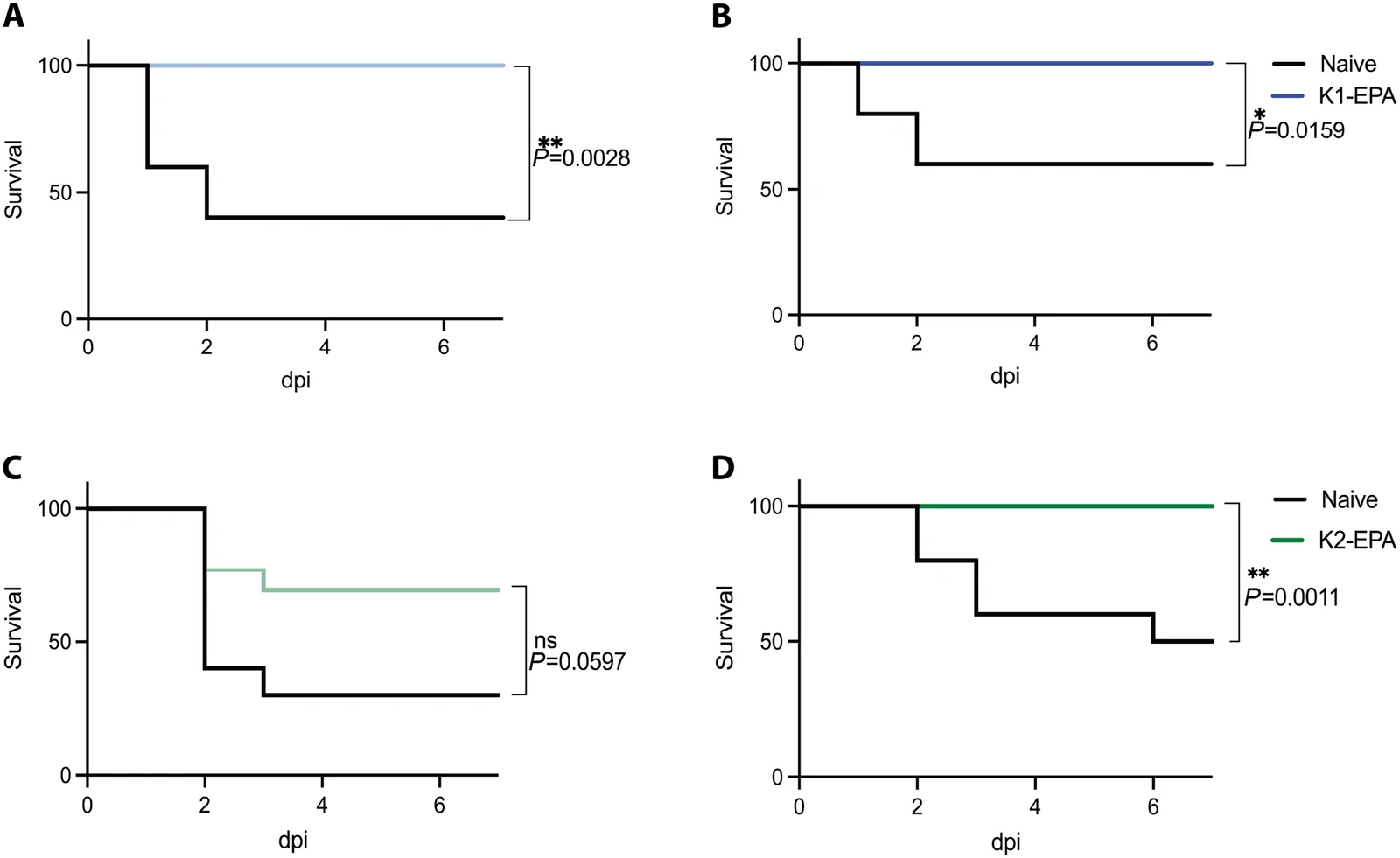
Six-week offspring remain protected from lethal challenge. Pups born to dams immunized with either K1-EPA or K2-EPA in 2 or 3 doses were weaned at 3 weeks of life and then challenged with lethal doses of *Klebsiella pneumoniae* at 6 weeks of life. (**A** and **B**) Pups born to dams immunized with K1-EPA were challenged with cKp120. (A) Pups born to dams immunized on the 2-dose regimen. (B) Pups born to dams immunized on the 3-dose regimen. (**C** and **D**) Pups born to dams immunized with K2-EPA were challenged with KR174. (C) Pups born to dams immunized on the 2-dose regimen. (D) Pups born to dams immunized on the 3-dose regimen. Naïve CD-1 female mice were used as control for this experiment. Statistical analyses were performed via log-rank (Mantel-Cox) test comparing naïve mice to pups born to dams immunized with K1-EPA or K2-EPA. dpi, days post infection. Pup numbers for K1 studies are *n* = 12–13 per group and for K2 studies are *n* = 10–17 per group. *P < 0.05, **P < 0.01; ns, not significant. Legend beside (B) applies to (A) and (B), and legend beside (D) applies to (C) and (D).

## DISCUSSION

*K. pneumoniae* is a troublesome pathogen that is increasingly difficult to treat given the rise in antibiotic resistance. Vaccination exemplifies an ideal strategy to both decrease the use of antibiotics and lower disease burden. Many populations would benefit from a *K. pneumoniae* vaccine, but perhaps none more so than neonates in low- and middle-income countries. The disease burden of *K. pneumoniae* neonatal sepsis continues to increase and has become the leading cause of infectious deaths in children under the age of five (*2*, *3*). An initial hurdle in vaccine design is choosing the appropriate target antigen. *K. pneumoniae* expresses two prominent surface polysaccharides: capsular polysaccharide (K-type) and O-antigen (O-type) of lipopolysaccharide. Several studies have demonstrated the immunogenicity of both targets (*7*–*9*, *19*–*22*) in animal models and humans. Further, much recent work has highlighted the distribution of K- and O-types in neonatal sepsis to better inform vaccine design (*23*). These recent advances, in addition to animal model development for therapeutic efficacy studies, bring the feasibility of a *K. pneumoniae* vaccine within reach.

K2 is one of the most prominent capsular types associated with *K. pneumoniae* neonatal sepsis (*23*), whereas K1 strains are rarely associated. K1, however, is often expressed by hypervirulent *K. pneumoniae* strains and may be an attractive addition to any *K. pneumoniae* polyvalent vaccine in development to expand its target population. Here, we demonstrate that each monovalent antigen is immunogenic and protective when delivered maternally in bioconjugate form, but ultimately response to each antigen should be evaluated when delivered in a polyvalent formulation with other antigens. Particularly intriguing for future studies is the simultaneous combination of K- and O-antigens in a vaccine and the relative synergism or antagonism that may result. We now have the tools to tackle and scrutinize these issues.

In this present work we explored two different dosing regimens. We found promising protection from both capsular antigens whether dams were vaccinated twice or thrice. Despite small differences in transferred capsule-specific IgG to pups, the subsequent protection data suggest that a two-dose vaccination regimen may be sufficient for protection from *Klebsiella* in this model. Clinically, a single dose immunization of pregnant women would be ideal given the difficulty of patient follow-up and healthcare access. This could be further explored in animal models; however, findings may not translate as mice are bred in a controlled environment in which they may never encounter *Klebsiella* antigens prior to immunization. In humans, however, the possibility exists that the immune system may have already been exposed to specific *Klebsiella* antigens and thus even a single dose vaccination during pregnancy may induce a natural booster response.

While this study describes an important murine model for the testing of various maternal immunizations to prevent *K. pneumoniae* neonatal sepsis, it is not without its limitations. In this preliminary work, pups were challenged at DOL14. Most neonatal sepsis cases occur in the first week of life (*24*) and thus future model iterations should consider pup challenge prior to one week of life to better recapitulate human disease. Secondly, while the two classical isolates used in this current study are representative in that they are classical (not hypervirulent isolates), they were not originally isolated from neonates. An optimal model would employ true neonatal sepsis isolates recovered from babies and representative of circulating isolates. Realistically a panel of isolates spanning the various antigens being considered for a vaccine would be included. Thirdly, we challenged pups via IP administration of *K. pneumoniae* which quickly results in bacteremia, bypassing the gastrointestinal (GI) tract. It is thought that most cases of neonatal sepsis originate in the GI tract prior to a translocation event which results in bacteremia (*25*). The presented model circumvents GI tract colonization and translocation which may or may not be affected by maternal vaccination. Finally, this model evaluates pups born after a full gestation; however, a high proportion of *K. pneumoniae* neonatal sepsis cases occur in premature infants (*26*). It may be that placental transfer of maternal IgG is not sufficient in premature neonates to ensure protection, which may require passive antibody administration or other preventative therapeutics.

The murine model presented in this current work may also be useful outside of solely informing vaccine efficacy. It provides a tool to explore many unanswered questions in the field such as the contribution of maternal antibodies delivered through the breastmilk versus placenta. We have begun to address these questions here by developing a method to collect murine breastmilk from lactating dams; however, further experiments could better inform the relative contributions. In this study we were unable to identify polysaccharide-specific IgA in the murine breastmilk. This is in stark contrast to what may be expected in human breastmilk. A recent review highlights the differences between mouse and human breastmilk composition: human breastmilk can contain up to 90% IgA and less than 5% IgG whereas murine breastmilk contains approximately 75% IgG (*27*). This harmonizes with our findings of primarily polysaccharide-specific IgG in vaccinated dam breastmilk. Additionally, mice have a hemotrichorial placenta containing three trophoblast layers, whereas human have a hemomonochorial placenta (*27*). Such differences between human and murine placental anatomy and breastmilk composition make it necessary to translate any murine model findings using human studies. Lastly, this model can serve as a means to understand how maternal vaccination affects bacterial colonization in dams and pups.

In summary, we have developed a murine model of maternal immunization and *K. pneumoniae* neonatal sepsis. These experiments have demonstrated the ability of K1- and K2-bioconjugate vaccines to protect offspring of vaccinated dams from lethal *K. pneumoniae* infection. Future studies will expand this work with model improvements and a characterized panel of *K. pneumoniae* neonatal sepsis strains, creating the groundwork to evaluate a variety of *K. pneumoniae* vaccine platforms and formulations.

## MATERIALS AND METHODS

### Bacterial strains and growth conditions

The *K. pneumoniae* human isolates used in this study were cKp120 (K1:O1αβ,2β) and KR174 (K2:O1αβ,2α), both of which have been previously described (*8*, *28*). Glycoengineered strains of *E. coli* were used for ELISA analyses; they were produced and grown as previously described (*7*). All *K. pneumoniae* isolates used for murine infections were grown statically in Luria-Bertani Broth at 37°C for 16 hours.

### Conjugate vaccine production

Bioconjugate vaccines were constructed using the K1 or K2 capsular polysaccharides of *K. pneumoniae* conjugated to a genetically inactivated variant of the EPA carrier protein (exotoxin A from *P. aeruginosa*) containing two PglS sequons in glycoengineered *E. coli*. K1-EPA and K2-EPA were produced, grown, and purified as previously described (*7*, *8*). Final bioconjugate polysaccharide structures were verified by both mass spectrometry and nuclear magnetic resonance spectroscopy, as previously described (*7*, *10*).

### Murine stool sequencing

Fecal samples were extracted using the MagAttract Powersoil Pro kit (Qiagen) on the KingFisher Apex. Nextera library preparation and sequencing were performed on the NovaSeq X Plus. Sequencing adapters were trimmed with Trimmomatic (v0.39) (*29*) with the following parameters: ILLUMINACLIP:NexteraPE-PE.fa:2:30:8:1:true, SLIDINGWINDOW:4:15, LEADING:10, TRAILING:10, MINLEN:60. Sequencing reads mapping to the mouse genome were removed with Deconseq (v0.4.3) (*30*). Reads were subsequently re-paired and filtered with the BBMap suite (v.39.01) (*31*) repair.sh script with the –tossbrokenreads flag. Pre- and post-processed read quality for each sample was assessed using FastQC (v0.12.1) (*32*). Bacterial taxonomic profiling was performed using MetaPhlAn (v4.2.4) (*33*) with the –skip_unclassified_estimation flag. Relative abundance stack bar plots were generated in R (v4.4.2).

### Murine immunizations

All murine experiments and immunizations complied with ethical regulations for animal testing and research at Washington University School of Medicine and were approved by the IACUC committee (#23–0300). Eight-week-old male and female CD-1 outbred mice (Charles River Laboratories) were mated. At time of mating (day 0 of mating) female mice (dams) were subcutaneously injected with 100 μl of a vaccine formulation. Dams in the 2-dose immunization group were boosted two weeks later (day 14 of mating). Dams in the 3-dose immunization group were boosted 1 and 2 weeks after the initial prime (7 and 14 days of mating). The vaccination groups were as follows: 2-doses of K1-EPA, K2-EPA or EPA; 3-doses of K1-EPA, K2-EPA, or EPA. All capsule vaccination groups received 1 μg of vaccine based on total polysaccharide content. All vaccines were formulated with Alhydrogel 2% aluminum hydroxide gel (InvivoGen) at a 1:9 ratio (50 μl vaccine to 5.5 μl adjuvant in 44.5 μl sterile PBS). Sera was collected from dams on days 0, 7, 14, and 21. Parturition occurred around day 21 and serum was collected from offspring (pups) on days indicated up to 6 weeks post birth. Serum was collected from dams and pups via mandibular bleed.

### Enzyme-linked immunosorbent assays

ELISAs were carried out as previously described (*8*). Briefly, 96-well ELISA plates were coated overnight with specified bacterial isolates in sodium carbonate buffer. After coating, wells were washed with 0.05% PBS-Tween-20 (PBS-T) and blocked with 1% bovine serum albumin in PBS. Mouse serum was diluted 1:100 in blocking buffer and added to wells, followed by washing, and the addition of HRP-conjugated secondary antibodies; 1:5000 for anti-mouse IgG (Biolegend 405306) and 1:2000 for anti-mouse IgA (Life Technologies 62–6720). Plates were washed and developed measuring absorbance at 450 nm using a microplate reader (Bio-Tek). Total antibody concentration was determined using a standard curve. All wells were normalized to blank wells treated identically to the sample wells without receiving primary antibody to assess assay background level. All graphs were generated using GraphPad Prism version 10.

### Murine breastmilk collection

Breastmilk was collected from dams around DOL eight of the offspring (*34*). Dams were separated from pups for no longer than one hour while pups were kept warm using heating elements. Dams were administered 200 μl of oxytocin (Bimeda, 20 USP units/ml) via intraperitoneal injection. Dam nipples were sterilized using alcohol and breastmilk was collected using a modified Lansinoh Signature Pro Double Electric breast pump. The pump was modified by attaching the plastic pump tubing to the needle side of 20-gauge needle. The back end of the needle was used to attach the nipple to suction and pump breastmilk for no more than 7 minutes per dam.

### Murine challenges

*K. pneumoniae* isolates were administered via intraperitoneal injection to induce neonatal sepsis. Pups were challenged at DOL 14 for most experiments and 6 weeks post birth for the protection longevity study. cKp120 was administered at 5 × 10^6^ CFU in 50 μl and KR174 at 1 × 10^8^ in 50 μl. Pup survival was monitored daily for seven days. Pairwise survival differences were determined by the Mantel-Cox log-rank test. All graphs and statistics were generated using GraphPad version 10.
