# Supplementary material for: Maternal vaccination with capsular polysaccharide bioconjugates protects against murine Klebsiella pneumoniae neonatal sepsis
Source: Sci Adv. 2026 Oct 14;12(42):eaeh8222. doi: 10.1126/sciadv.aeh8222 (PMC13665941; doi:10.1126/sciadv.aeh8222)
Supplement: Supplementary file 1 [file sciadv.aeh8222_sm.pdf]

Science Advances
AAAS

Supplementary Materials for

Maternal vaccination with capsular polysaccharide bioconjugates protects against murine *Klebsiella pneumoniae* neonatal sepsis

Paeton L. Wantuch *et al.*

Corresponding author: David A. Rosen, rosend@wustl.edu

Sci. Adv. **12**, eaeh8222 (2026)
DOI: 10.1126/sciadv.aeh8222

This PDF file includes:

Figs. S1 to S3

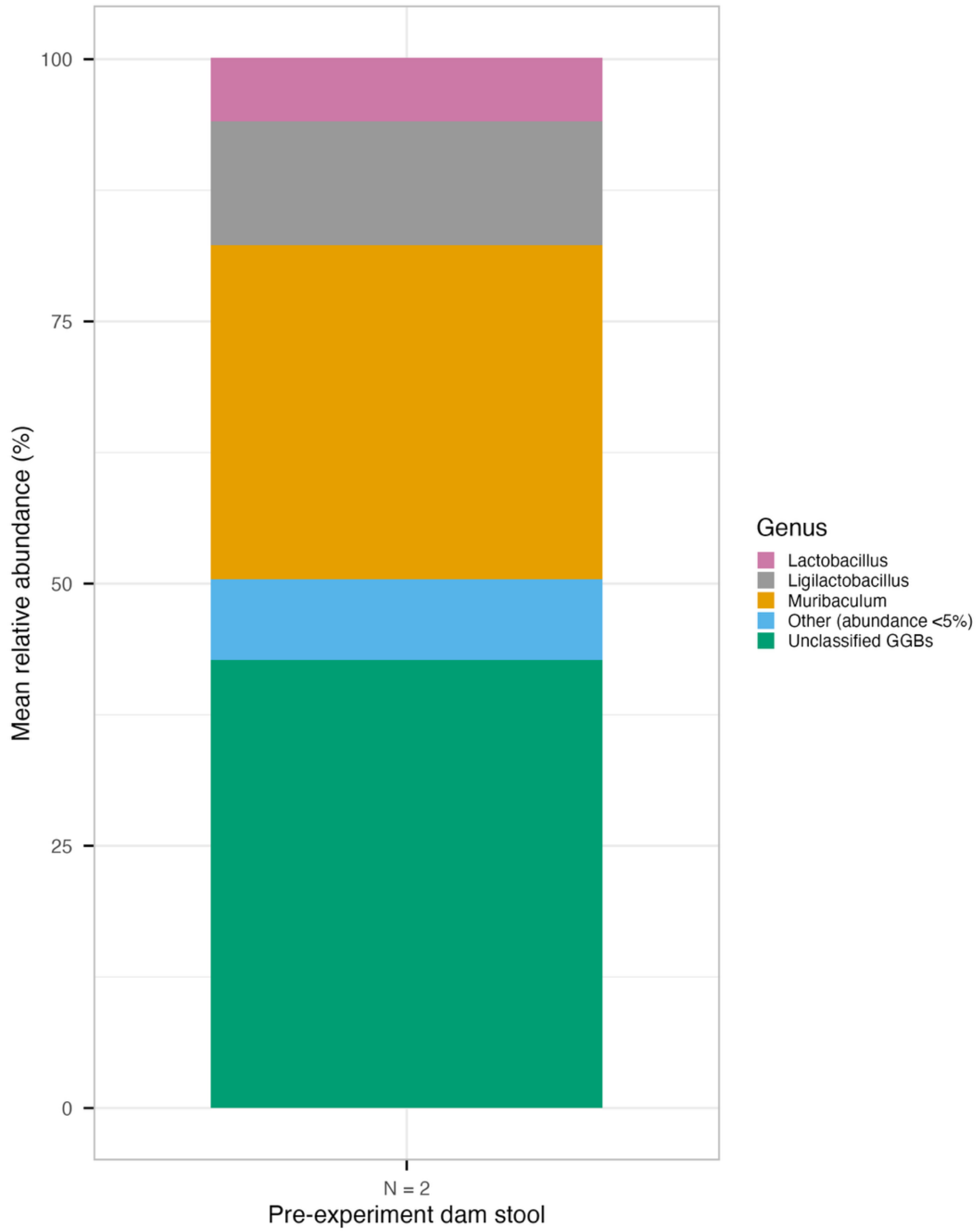

Fig. S1: **Stool shotgun metagenomic sequencing of CD-1 female mice prior to mating and infection.** Stool samples were collected from CD-1 female mice. Per-sample genus-level taxonomic profiling was performed using MetaPhlAn4. Stacked bar plot displays the mean relative abundance for N = 2 mice (one fecal sample each). GGB = Genus-level Genome Bin; unclassified GGBs represent yet to be characterized genera.

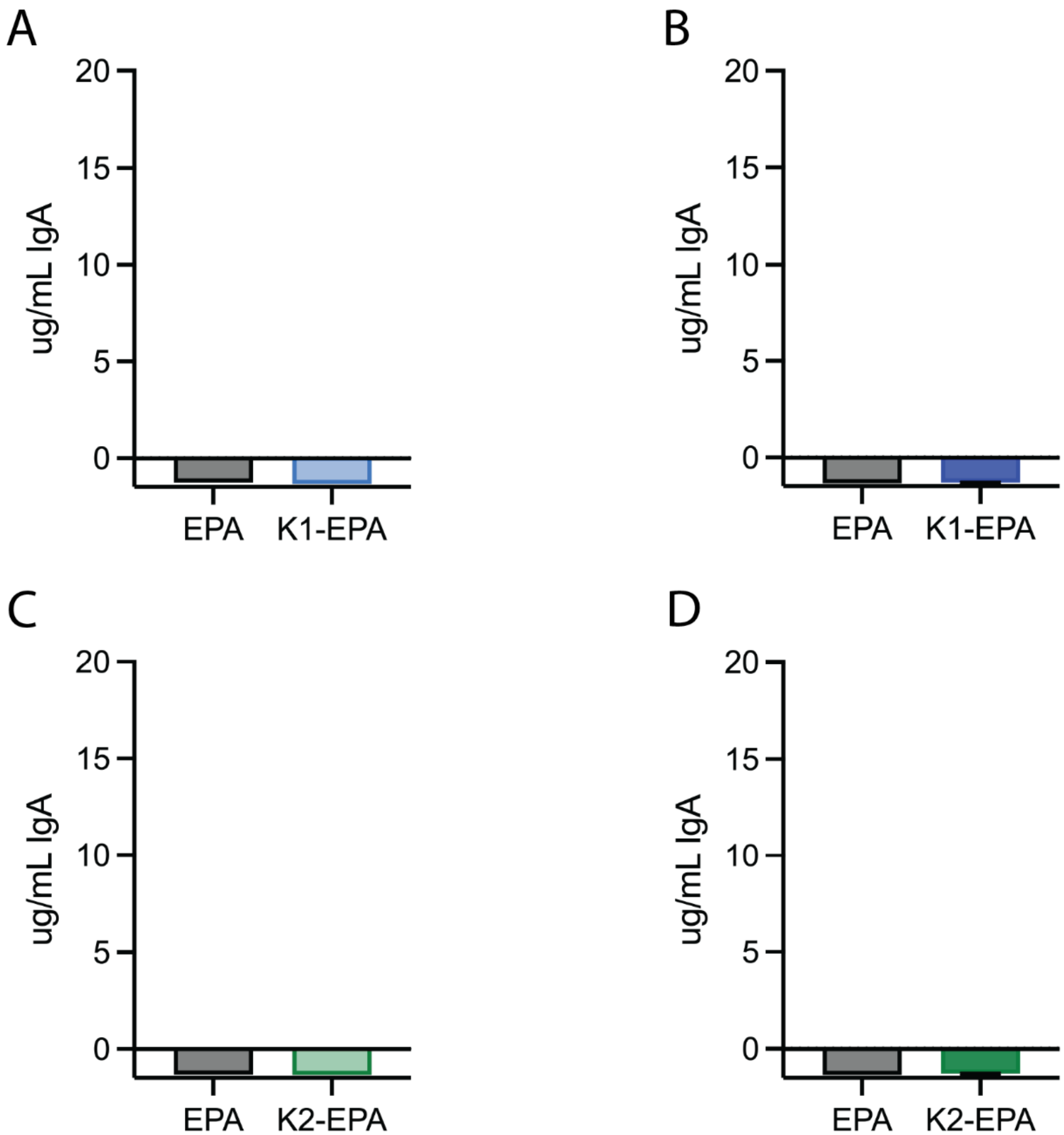

Fig. S2: **Polysaccharide-specific IgA antibodies in the breastmilk.** Breastmilk was collected from lactating dams after immunization with either K1-EPA (blue) or K2-EPA (green) conjugates. Polysaccharide-specific IgA concentrations were detected as measured by ELISA on plates coated with glycoengineered *E. coli* expressing A,B) K1 polysaccharide or C,D) K2 polysaccharide. Dams were immunized with either 2 doses (A,C light blue or green) or 3 doses (B,D dark blue or green) of K1-EPA or K2-EPA conjugate vaccines or EPA (black) as control. Graphs display immunoglobulin concentrations as calculated from a standard curve. Dam numbers are n=1-2 per group.

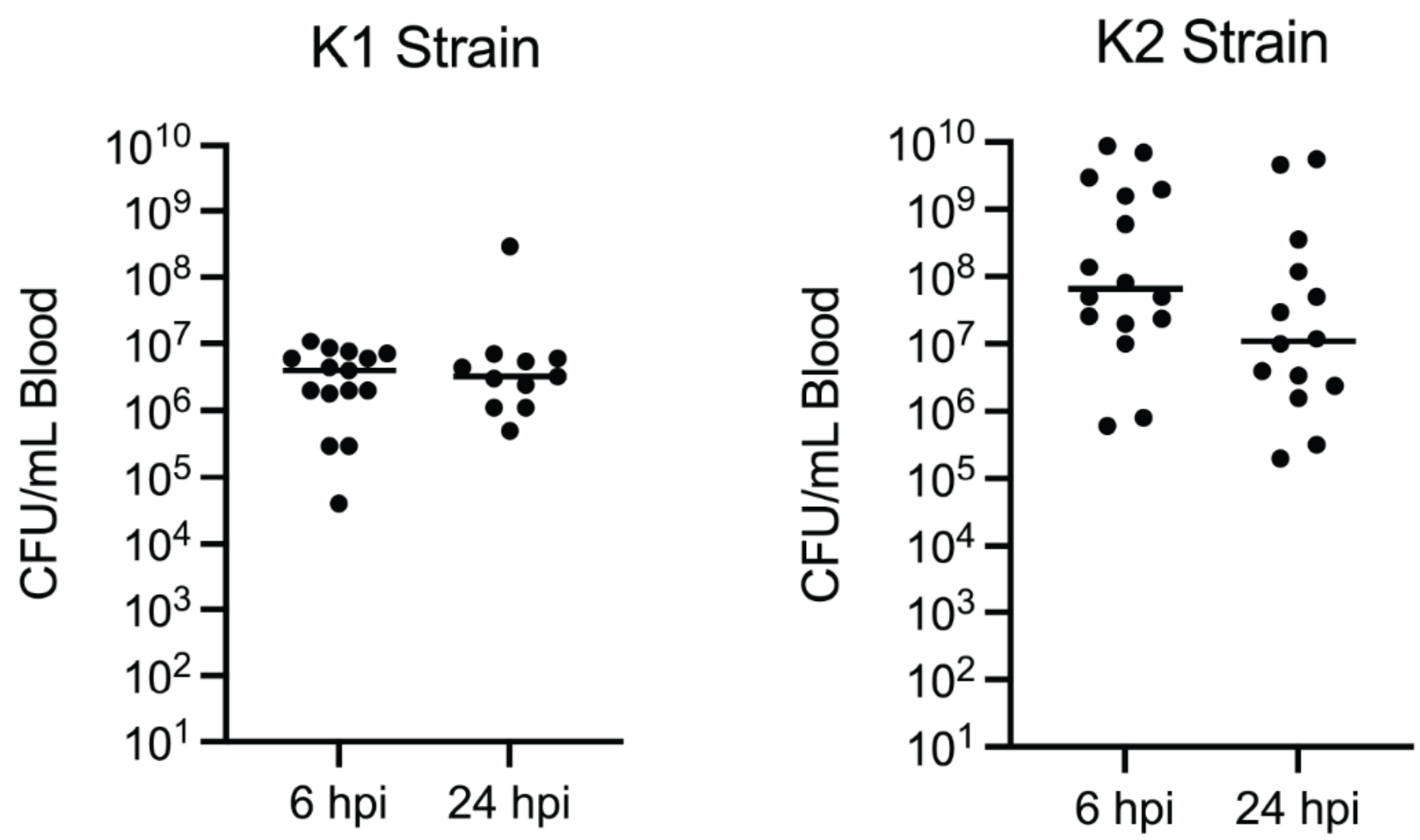

Fig. S3: **Bacterial burden in the blood measured 6 and 24 hours post infection.** Bacteria were administered via intraperitoneal injection on pup day of life 14 and blood was collected via mandibular bleed at the given subsequent timepoints. Pups were born from unvaccinated dams. Blood was serially diluted and plated to enumerate number of colony forming units. Horizontal bars represent geometric means.
